# Donepezil ameliorates fatigue and depression in PASC patients with HHV-6B SITH-1-induced acetylcholine deficiency

**DOI:** 10.3389/fphar.2026.1807203

**Published:** 2026-06-04

**Authors:** Naomi Oka, Kensuke Nakamura, Koichi Hirahata, Azusa Ishii, Kazuma Yamakawa, Kenya Ie, Tadahiro Goto, Kazuya Shimada, Shigeki Fujitani, Kazuhiro Kondo

**Affiliations:** 1 Department of Virology, The Jikei University School of Medicine, Tokyo, Japan; 2 Department of Critical Care Medicine, Yokohama City University Hospital, Yokohama, Kanagawa, Japan; 3 Hirahata Clinic, Tokyo, Japan; 4 Department of Emergency and Critical Care Medicine, Osaka Medical and Pharmaceutical University, Takatsuki, Osaka, Japan; 5 Department of General Internal Medicine, Kawasaki Municipal Tama Hospital, Kawasaki, Kanagawa, Japan; 6 TXP Medical Co. Ltd., Tokyo, Japan; 7 Department of Emergency and Critical Care Medicine, St. Marianna University School of Medicine, Kawasaki, Kanagawa, Japan; 8 Department of Fatigue Science, The Jikei University School of Medicine, Tokyo, Japan

**Keywords:** acetylcholine, depression, donepezil, fatigue, human herpesvirus 6B, long-COVID, post-acute sequelae of SARS-CoV-2 infection, SITH-1

## Abstract

**Introduction:**

The pathogenesis of post-acute sequelae of SARS-CoV-2 infection (PASC) remains poorly understood, and no effective treatment has been established. Reactivation of latent herpesviruses, particularly human herpesvirus 6B (HHV-6B), has been proposed as a possible contributor to the neuropsychiatric symptoms observed in PASC. SITH-1, a latency-associated protein expressed during HHV-6B reactivation in olfactory bulb astrocytes, induces specific antibody responses that can be detected in peripheral blood. Importantly, SITH-1 has also been identified as a risk factor for depression, suggesting a mechanistic link between HHV-6B reactivation and the development of neuropsychiatric symptoms.

**Methods:**

We measured serum anti-SITH-1 antibody titers in 156 PASC patients and compared them to healthy controls. In this PASC cohort, neuropsychiatric symptoms were assessed using numerical rating scales. In parallel, we developed a mouse model in which SITH-1 was transiently expressed in the olfactory bulb to assess its impact on brain function and behavior. We also conducted a subgroup analysis of a previously reported randomized clinical trial (RCT) of donepezil, stratifying PASC patients by anti-SITH-1 antibody status.

**Results:**

Anti-SITH-1 antibody positivity was observed in 62.8% of PASC patients, a significantly higher proportion than in controls. Seropositive patients exhibited more severe fatigue and depressive symptoms. In the mouse model, SITH-1 expression led to reduced acetylcholine production and depression-like behavior, both of which were ameliorated by donepezil. In the clinical trial subgroup of 73 PASC patients, 71.7% were seropositive for anti-SITH-1 antibodies. Among these individuals, donepezil significantly improved fatigue and depression scores, as measured by the Chalder Fatigue Scale and the depression subscale of the Hospital Anxiety and Depression Scale (HADS).

**Conclusion:**

These findings suggest that HHV-6B reactivation in the olfactory bulb, as indicated by anti-SITH-1 antibody titers, may contribute to fatigue and depression in a subset of PASC patients. Donepezil may be effective in this subgroup, and these findings support the use of anti-SITH-1 antibody titers as a companion diagnostic marker for targeted treatment in PASC.

## 1 Introduction

Post-acute sequelae of SARS-CoV-2 infection (PASC), also known as long COVID, refer to persistent health effects that occur after the acute phase of infection. PASC affects approximately 5%–10% of individuals previously infected with SARS-CoV-2 and has become a major global health and social concern. However, its underlying etiology remains unclear, and no effective pharmacological treatment has been established to date (Choutka et al., 2022; Davis et al., 2023; Al-Aly and Topol, 2024; Greenhalgh et al., 2024; Peluso and Deeks, 2024).

The World Health Organization (WHO) defines PASC as the continuation or onset of new symptoms 3 months after the initial SARS-CoV-2 infection, with symptoms lasting for at least 2 months and having no alternative explanation (Soriano et al., 2022; Thaweethai et al., 2023). As of 2025, between 80 and 400 million individuals worldwide are estimated to be affected (Wilson et al., 2025). While PASC symptoms vary widely (Davis et al., 2021; Ballering et al., 2022), fatigue, depression, brain fog, and other neuropsychiatric symptoms are particularly prevalent (Michelen et al., 2021; Taquet et al., 2021; Roessler et al., 2022; Sampogna et al., 2022; Kubota et al.,, 2023; Fernandez-de-Las-Peñas et al., 2024). Among these, fatigue and depression are especially debilitating, significantly impairing patients’ quality of life (QOL) and capacity to work (Lopez-Leon et al., 2022; Premraj et al., 2022; Walker et al., 2023; McLaughlin et al., 2025).

Although multiple hypotheses have been proposed regarding the pathogenesis of PASC, many aspects remain unresolved (Iwasaki and Putrino, 2023). Proposed mechanisms include persistent SARS-CoV-2 infection in tissues (Swank et al., 2023), immune damage (Ruf, 2024), decreased cortisol levels (Klein et al., 2023), complement dysregulation (Cervia-Hasler et al., 2024), and reduced serotonin (Wong et al., 2023). In addition, reactivation of latent Herpesviridae viruses, particularly human herpesvirus 6B (HHV-6B), has emerged as a strong candidate mechanism contributing to PASC (Prtajin and Ljubojević Hadžavdić, 2022; Su et al., 2022; Zubchenko et al., 2022; Altmann et al., 2023; Hashimoto, 2023; Klein et al., 2023; Vojdani et al., 2023; Butt et al., 2024). These observations highlight the likelihood that PASC is a multifactorial condition requiring elucidation of diverse pathophysiological mechanisms and development of targeted therapies tailored to individual subtypes.

To explore one such mechanism, we focused on the disruption of acetylcholine signaling in the context of SARS-CoV-2 infection (Lysenkov et al., 2021; Lykhmus et al., 2022; Nadwa et al., 2023; Oka et al., 2023). In a previous study, we demonstrated that a COVID-19 model mouse expressing the SARS-CoV-2 spike (S1) protein in the nasal cavity exhibited fatigue- and depression-like behavioral symptoms resulting from brain inflammation. This inflammation was associated with dysfunction of the cholinergic anti-inflammatory pathway, driven by reduced levels of brain acetylcholine. Administration of the acetylcholinesterase inhibitor like donepezil alleviated both the behavioral symptoms and brain inflammation, likely via restoration of cholinergic anti-inflammatory pathway (Oka et al., 2023). Based on these findings, we conducted a randomized clinical trial (RCT) to investigate the efficacy of donepezil in treating post-COVID-19 fatigue and psychological symptoms (Kawabata et al., 2024; Nakamura et al., 2025).

In that trial, however, donepezil did not show statistically significant efficacy, likely due to the inclusion of participants who either did not meet strict diagnostic criteria for PASC or had PASC arising from heterogeneous etiologies (Nakamura et al., 2025). This led us to hypothesize that a subgroup of patients might benefit from donepezil when stratified using relevant biomarkers. In particular, anti-SITH-1 antibody titers, which reflect reactivation of HHV-6B through expression of the latency-associated protein SITH-1, emerged as a promising biomarker for identifying patients who are responsive to treatment (Kobayashi et al., 2020).

HHV-6B typically causes roseola infantum during primary infection and then establishes lifelong latency (Ablashi et al., 2014). In humans, HHV-6B persists in two main cellular reservoirs. One is peripheral blood macrophages, where the latency-associated gene H6LT is expressed during both latency and reactivation (Kondo et al., 2002). Reactivation in macrophages, typically under immunosuppressed conditions, leads to systemic infection and can be detected by elevated HHV-6B DNA levels in blood (Kondo et al., 2002).

The second site of latency is the brain, particularly astrocytes in the olfactory bulb (Donati et al., 2005). In this compartment, the SITH-1 protein is expressed during both latency establishment and reactivation (Kobayashi et al., 2020). Although olfactory bulb tissue is not readily accessible in humans, reactivation in this site can be inferred from elevated serum anti-SITH-1 antibody titers (Kobayashi et al., 2020). Because HHV-6B reactivation in the olfactory bulb can occur even in individuals with normal immune function, the reactivated virus remains localized near the olfactory bulb and does not lead to systemic HHV-6B infection.

A link between HHV-6B reactivation and depression has been reported (Prusty et al., 2018; Vojdani et al., 2024), particularly involving SITH-1 expression (Kobayashi et al., 2020; 2024). Elevated anti-SITH-1 antibody titers, as a proxy for SITH-1 expression, have been detected in approximately 80% of patients with major depressive disorder (Kobayashi et al., 2020). SITH-1 expression in the olfactory bulb has been linked to the pathogenesis of depression (Kobayashi et al., 2020). Notably, fatigue and depression, key features of PASC, overlap with core symptoms of major depressive disorder (Demyttenaere et al., 2005; Sibitz et al., 2010). Based on these observations, we hypothesized that anti-SITH-1 antibody titers could serve as a stratification biomarker in a randomized trial evaluating the efficacy of donepezil in PASC-related neuropsychiatric symptoms.

In this study, we report that expression of SITH-1, a known risk factor for depression, is frequently observed in patients with PASC. We further examine its potential contribution to neuropsychiatric symptoms in PASC and demonstrate the therapeutic effect of donepezil in a mouse model expressing SITH-1. Finally, we perform a subgroup analysis of our previous RCT to evaluate the efficacy of donepezil in PASC patients who are seropositive for anti-SITH-1 antibodies, reflecting HHV-6B reactivation and SITH-1 expression in the olfactory bulb.

## 2 Methods

### 2.1 Participants in the biomarker study: PASC patients and normal controls

To identify a biomarker capable of stratifying patients for subgroup analysis in a clinical trial, we enrolled a total of 156 patients with post-acute sequelae of SARS-CoV-2 infection (PASC) at Hirahata Clinic (Tokyo), including 45 men and 111 women. All patients were diagnosed according to the World Health Organization (WHO) criteria for post-COVID-19 condition. Symptom severity was assessed for individual PASC symptoms using a numerical rating scale (NRS) ranging from 0 (no symptom) to 10 (worst imaginable severity).

As healthy normal controls (NCs), 36 volunteers (16 men and 20 women) with no history of SARS-CoV-2 infection were recruited by Soiken Inc. through advertisements. To ensure mental and physical health, all NCs were screened using the Beck Depression Inventory (BDI) and the Chalder Fatigue Scale (CFS), and individuals with elevated scores on either scale were excluded. Participants were also excluded if they were currently taking chronic medications or vitamin supplements.

All procedures involving human subjects were conducted in accordance with the Declaration of Helsinki and were approved by the Ethics Committees of the Jikei University School of Medicine, Soiken Inc., and Soiken Clinic. Written informed consent was obtained from all participants prior to their inclusion in the study.

### 2.2 Participants in the randomized clinical trial (RCT) and subgroup analysis

A multicenter, randomized, placebo-controlled, double-blind clinical trial of donepezil for post-COVID-19 fatigue and neuropsychiatric symptoms was conducted in Japan between 14 December 2022, and 31 March 2024. The trial protocol has been published previously (Kawabata et al., 2024). In the original RCT, participants with comorbid psychiatric disorders or concomitant psychiatric medications were excluded at enrollment, as specified in the protocol. Briefly, 120 eligible participants were enrolled and randomly assigned in a 1:1 ratio to receive donepezil or placebo for 3 weeks, and patient-reported outcomes were assessed at 3 and 8 weeks after treatment initiation. Ten individuals withdrew or were lost to follow-up, resulting in 110 patients (55 in the donepezil group and 55 in the placebo group) being included in the final efficacy analysis. Baseline characteristics (e.g., age, sex, symptom severity) did not differ significantly between groups. As reported in the primary analysis (Nakamura et al., 2025), donepezil did not demonstrate a statistically significant effect in the overall cohort, with a mean difference in CFS score at 3 weeks of 0.34 (95% CI, −2.23 to 2.91; P = 0.79).

In the present study, we conducted a retrospective subgroup analysis using serum samples collected during the RCT to investigate the relationship between anti-SITH-1 antibody titers and treatment efficacy. This subgroup analysis was conducted *post hoc* using stored serum samples from the previously reported RCT and was not pre-specified in the original trial protocol. Accordingly, results from the subgroup analyses should be interpreted as exploratory and hypothesis-generating. Of the 110 participants, 73 patients (35 men and 38 women) were included in the subgroup analysis as PASC cases, based on the WHO concept of symptom persistence for at least 2 months. For this subgroup analysis, PASC was operationally defined as persistent fatigue lasting at least 60 days after SARS-CoV-2 infection. These 73 patients were analyzed according to their original randomized, double-blind treatment assignment to placebo (n = 35; 12 men, 23 women) or donepezil (n = 38; 16 men, 22 women).

Patient-reported outcomes were assessed using validated self-report questionnaires, including the 11-item Chalder Fatigue Scale (CFS), the Hospital Anxiety and Depression Scale (HADS), and the Patient Health Questionnaire-9 (PHQ-9). Assessments were conducted online at 3 and 8 weeks after initiation of treatment. Treatment efficacy was evaluated by calculating the change in total scores from baseline, with more negative values indicating greater symptom improvement. Medication adherence was monitored by counting unused medication and returned packaging, and adherence below 80% was considered a protocol deviation.

Among the 73 patients included in the subgroup analysis, 52 participants (71.2%; 18 men and 34 women) were seropositive for anti-SITH-1 antibodies. Treatment efficacy was compared between seropositive and seronegative groups.

### 2.3 Anti-SITH-1 antibody titration

Detection of anti-SITH-1 antibodies was performed using an indirect immunofluorescence assay, as previously described (Kobayashi et al., 2020), with a modified method for titer quantification.

To generate the antigen for staining, a fusion construct comprising SITH-1, a flexible linker ((Gly-Gly-Gly-Gly-Ser)_5_), and CAML was cloned into the pFLAG-CMV-5a vector (Sigma-Aldrich). The amino acid sequence of SITH-1 is available under GenBank Accession Number AOC08123.1. The resulting plasmid (pCMV-SITH-CAML) was transfected into HEK293T cells cultured on Lab-Tek chamber slides (Nunc) using Lipofectamine LTX (Thermo Fisher Scientific).

Plasma samples were diluted 1:80 in PBS containing 2% BSA and 0.05% Tween 20, and mixed with a 1:200 dilution of anti-CAML mouse monoclonal antibody. The mixture was applied to the slides and incubated at 37 °C for 1 h. After washing with PBS/0.05% Tween 20, cells were incubated with secondary antibodies, Alexa Fluor 488–conjugated goat anti-human IgG (RRID: AB_2534080) and Alexa Fluor 594–conjugated goat anti-mouse IgG (RRID: AB_2534073) (Thermo Fisher Scientific), diluted 1:200 in PBS/2% BSA/0.05% Tween 20 at 37 °C for 30 min. Slides were washed again and mounted with cover glasses.

Fluorescence was visualized using an Olympus BX51 microscope equipped with a DP-73 CCD camera. Image analysis was conducted using ImageJ (NIH). The anti-SITH-1 Antibody titers were calculated using the following

$$\text{Titer}=\frac{{\text{AGFI}}_{SITH‐CAML}-{\text{AGFI}}_{\text{untransfected}}}{{\text{ARFI}}_{SITH‐CAML}-{\text{ARFI}}_{\text{untransfected}}}$$

Here, AGFI (average green fluorescence intensity) represents the fluorescence intensity of bound human IgG and reflects the amount of anti-SITH-1 antibodies present in the serum sample. ARFI (average red fluorescence intensity) represents the fluorescence intensity of bound anti-CAML antibody and serves as an indicator of the expression level of the antigen, namely, the SITH-CAML fusion protein, in the transfected cells.

### 2.4 Determination of SARS-CoV-2 strain

The infecting SARS-CoV-2 strain was estimated based on the predominant circulating variant in Tokyo, the region where participants resided, at the time of their COVID-19 onset. Specifically, infections were attributed to the wild-type (B.1.1) strain until February 2021, the Alpha variant (B.1.1.7) from March to June 2021, the Delta variant (B.1.617.2) from July to September 2021, and the Omicron variant (B.1.1.529 and sublineages) from October 2021 onward.

### 2.5 Animals

All animal experiments were conducted using male 8-week-old C57BL/6NCrSlc mice (Sankyo Laboratories, Japan). Mice were housed under standard laboratory conditions with a 12-h light/dark cycle (lights on at 8:00 a.m.), ambient temperature maintained at 24 °C ± 1 °C, and *ad libitum* access to food and water. All procedures were approved by the Animal Care and Use Committee of the Jikei University School of Medicine and conducted in accordance with institutional guidelines for animal experimentation.

### 2.6 Viruses and cells

Human embryonic kidney cell lines HEK293A and HEK293T (derived from female fetal tissue) were cultured in Dulbecco’s Modified Eagle Medium (DMEM) supplemented with 10% fetal bovine serum (FBS) at 37 °C in a humidified atmosphere containing 5% CO_2_.

The SARS-CoV-2 S1-expressing adenoviral vector was constructed as previously described (Oka et al., 2023), using the Adenovirus Dual Expression Kit (Takara Bio) according to the manufacturer’s instructions. Briefly, the SARS-CoV-2 S1 gene was cloned into the adenoviral cosmid vector pAxCAwtit2 (SARS-CoV-2-S1/pAxCAwtit2), and the recombinant adenovirus was generated by transfecting this construct into HEK293A cells.

The HHV-6B SITH-1-expressing adenoviral vector was generated as previously reported (Kobayashi et al., 2020), using the Adenovirus Expression Vector Kit (Takara Bio). The glial fibrillary acidic protein (GFAP) promoter was obtained from the pGfa2Lac plasmid (kindly provided by Dr. Kazuyoshi Ikuta). The GFAP promoter and the PCR-amplified SITH-1 gene were cloned into an adenoviral cosmid to construct Ad-GFAP-SITH1. HEK293A cells were co-transfected with Ad-GFAP-SITH1 and the helper cosmid (pAxcwit2) to produce recombinant virus.

All recombinant adenoviruses were propagated in HEK293A cells and purified using the Adeno-X Virus Purification Kit (Takara Bio). Viral titers were quantified using the Adeno-X Rapid Titer Kit (Takara Bio).

### 2.7 Nasal inoculation of adenoviral vectors

Eight-week-old male C57BL/6 mice were anesthetized with isoflurane prior to intranasal administration. Each adenoviral vector was diluted in sterile water (rather than isotonic buffer). A 20-μL drop of the viral solution, containing 1–2 × 10^9^ plaque-forming units (pfu)/mL, was placed at the entrance of each mouse’s nasal cavity. The solution was inhaled passively through spontaneous respiration.

### 2.8 Drug administration

The acetylcholinesterase inhibitor donepezil (FUJIFILM Wako) was administered via drinking water at a concentration of 40 mg/L, corresponding to an approximate dose of 4 mg/kg/day. Treatment began on the same day as adenovirus inoculation and continued throughout the experimental period.

### 2.9 Animal behavior tests

Seven days after intranasal inoculation with S1-Ad or Vector-Ad, mice underwent behavioral testing to evaluate fatigue- and depression-like symptoms.

In the tail suspension test (TST), immobility time over a 10-min period was recorded and analyzed using TailSuspScan software (CleverSys Inc.). In the Y-maze test, the number of arm entries during an 8-min session was measured using TopScan software (CleverSys Inc.).

Twenty-four hours after completion of the behavioral tests, mice were euthanized, and brain tissues were collected for real-time PCR and immunohistochemical analysis.

### 2.10 Real-time PCR

Total RNA was extracted from mouse tissues using the RNeasy Mini QIAcube Kit (Qiagen), and reverse transcription was performed using the PrimeScript RT Reagent Kit (Takara Bio) to synthesize complementary DNA (cDNA). Quantitative real-time PCR was carried out using Premix Ex Taq (Perfect Real Time) (Takara Bio) and the QuantStudio 3 Real-Time PCR System (Thermo Fisher Scientific).

The thermal cycling conditions were as follows: initial denaturation at 95 °C for 30 s, followed by 45 cycles of denaturation at 95 °C for 5 s and annealing/extension at 60 °C for 31 s. Data were analyzed using QuantStudio Design and Analysis Software (Thermo Fisher Scientific).

TaqMan Gene Expression Assays (Thermo Fisher Scientific) were used to quantify mRNA levels of the following genes: Mouse Il1b (IL-1β): Assay ID Mm00434228_m1, Mouse Chat (Choline acetyltransferase): Assay ID Mm01221880_m1, Mouse Fgf2 (basic FGF): Assay ID Mm01285715_m1, Eukaryotic 18S rRNA (endogenous control): Assay ID Hs99999901_s1.

Relative gene expression levels were normalized to 18S rRNA using the ΔΔCt method.

### 2.11 Immunohistochemistry

For immunohistochemical analysis, mice were euthanized and transcardially perfused with physiological saline followed by 10% neutral-buffered formalin (pH 7.4). The brains were harvested, fixed in formalin, embedded in paraffin, and sectioned. For immunofluorescence staining, paraffin-embedded sections were deparaffinized, subjected to antigen retrieval, and blocked with Image-iT FX Signal Enhancer (Thermo Fisher Scientific) for 30 min at room temperature.

Primary antibodies included anti–active caspase-3 (RRID: AB_443014), anti–doublecortin (DCX) (RRID: AB_732011), and anti–choline acetyltransferase (ChAT) (RRID: AB_2721842), all obtained from Abcam. Secondary antibody (Alexa Fluor 488 goat anti-rabbit IgG [H+L]) (RRID: AB_2576217) was obtained from Thermo Fisher Scientific. All antibodies were diluted using Can Get Signal® Immunostain Solution A (TOYOBO).

After staining, sections were mounted with coverslips and observed using a fluorescence microscope (BZ-9000, Keyence).

### 2.12 Quantification and statistical analysis

Normality of data distribution was assessed using the Shapiro–Wilk test. For comparisons between two groups, the nonparametric Mann–Whitney U test was used. For comparisons among multiple groups, one-way analysis of variance (ANOVA) followed by Fisher’s least significant difference (LSD) *post hoc* test was used for parametric data, while the Kruskal–Wallis test followed by Dunn’s *post hoc* test was used for nonparametric data. In the figures, red horizontal lines indicate either the median or mean value. A p-value of <0.05 was considered statistically significant.

The RCT subgroup analyses were *post hoc* and exploratory, and p values are reported as nominal. To address multiple testing, p values from placebo versus donepezil comparisons across three strata (overall, anti-SITH-1 positive, anti-SITH-1 negative) and two time points (weeks 3 and 8) were additionally adjusted using the Benjamini–Hochberg false discovery rate procedure within each questionnaire family. FDR-adjusted q values are provided in Supplementary Table S1.

For comparisons of baseline characteristics in Tables 1, 2, continuous variables, including age and post-onset period, were analyzed using the Mann–Whitney U test, and the categorical variable sex was analyzed using Fisher’s exact test. All statistical analyses were performed using Prism 8 (GraphPad Software) and BellCurve for Excel (Social Survey Research Information Co., Ltd.).

**TABLE 1:** Baseline characteristics of participants in the preliminary cohort.

Characteristic	Normal controls (n = 36)	Post-acute sequelae of SARS-CoV-2 infection (PASC) patients (n = 156)	*P* (NC vs. PASC)	anti-SITH-1-Ab negative PASC patients (n = 58)	anti-SITH-1-Ab positive PASC patients (n = 98)	*P* (anti-SITH-1 negative vs. positive)
Age (years) [median (IQR)]	42.5 (37–52)	43 (35–49)	0.775^1^	42.5 (32–49)	43 (36.8–49)	0.550^1^
Gender (%)	​	​	0.077^2^	​	​	0.466^2^
Male	16 (44)	45 (29)	​	19 (33)	26 (27)	​
Female	20 (56)	111 (71)	​	39 (67)	72 (73)	​
Post-onset period [median (IQR)]	-	502 (325–643)	​	486 (318–608)	512 (326–705)	0.303^1^

Baseline characteristics are shown for normal controls (NCs) and patients with post-acute sequelae of SARS-CoV-2 infection (PASC), and for PASC patients stratified by anti-SITH-1 antibody status. Age and post-onset period were compared using the Mann–Whitney U test, and sex was compared using Fisher’s exact test. Post-onset period was available only for PASC patients.

Tests used:

1 Mann-Whitney U test.

2 Fisher’s Exact Test.

**TABLE 2:** Baseline characteristics of PASC patients in the donepezil RCT subgroup analysis.

Characteristic	Total			SITH-1 positive			SITH-1 negative		
​	Placebo (n = 35)	Donepezil (n = 38)	*p*	Placebo (n = 26)	Donepezil (n = 26)	*p*	Placebo (n = 9)	Donepezil (n = 12)	*p*
Age (years) [median (IQR)]	45 (35–47)	46.5 (33.3–53.5)	0.270^1^	41.5 (33.8–47.5)	47 (34.8–55)	0.227^1^	45 (36–50)	45 (31.8–52.3)	>0.999^1^
Gender (%)	​	​	0.631^2^	​	​	0.382^2^	​	​	0.670^2^
Male	12 (34)	16 (42)	​	7 (27)	11 (42)	​	5 (56)	5 (42)	​
Female	23 (66)	22 (58)	​	19 (73)	15 (58)	​	4 (44)	7 (58)	​
Post-onset period [median (IQR)]	276 (196–314)	266 (193–311.3)	0.889^1^	268.5 (170.8–311)	258 (144.3–324.5)	0.775^1^	292 (236.5–323)	290 (263.8–305.3)	0.987^1^

Age and post-onset period were compared using the Mann Whitney U test, and sex was compared using Fisher’s exact test. Data are shown for the total cohort and stratified by anti-SITH-1 antibody status.

Tests used:

1 Mann-Whitney U test.

2 Fisher’s Exact Test.

## 3 Results

### 3.1 Elevated anti-SITH-1 antibody titers are frequently observed in PASC patients and are associated with more severe neuropsychiatric symptoms

**FIGURE 1:**
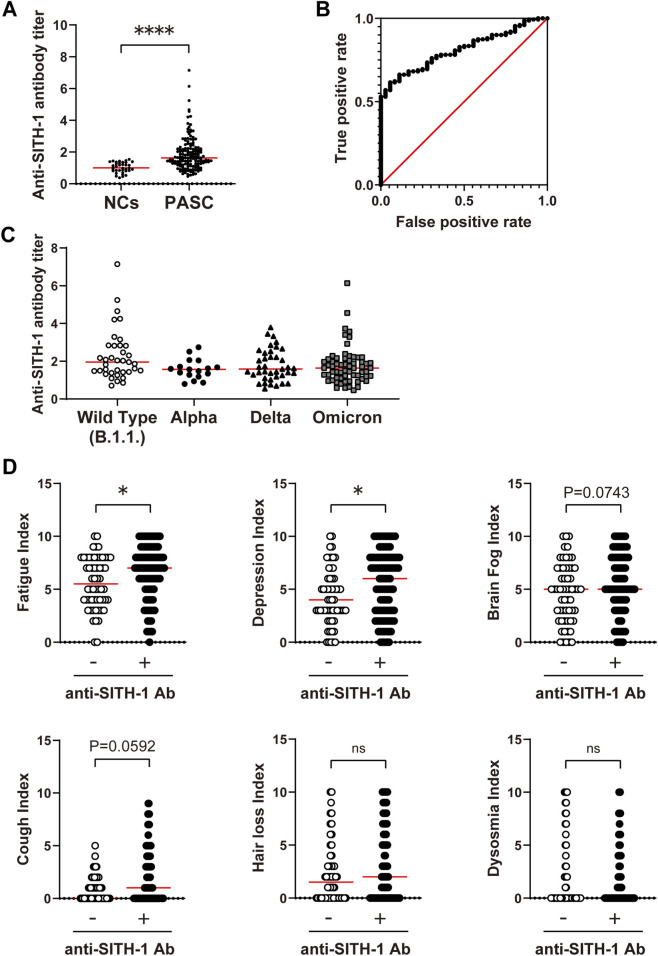
Elevated anti-SITH-1 antibody titers are frequently observed in PASC patients and are associated with more severe neuropsychiatric symptoms. **(A)** Serum anti-SITH-1 antibody titers in normal controls (NCs) with no history of SARS-CoV-2 infection and patients with PASC (NCs, n = 36; PASC, n = 156). Titers were normalized to the median value in NCs, which was set to 1. **(B)** Receiver operating characteristic (ROC) curve for anti-SITH-1 antibody titers distinguishing NCs and PASC patients. The threshold of 1.43 was used to define anti-SITH-1 antibody positivity. **(C)** Anti-SITH-1 antibody titers stratified by presumed SARS-CoV-2 variant based on the period of infection (wild type, n = 37; α, n = 17; δ, n = 39; ο, n = 63). **(D)** NRS scores (0–10) for individual PASC symptoms in anti-SITH-1 antibody seronegative (n = 58) and seropositive (n = 98) patients. Data are shown as individual values with red horizontal lines indicating the median. Statistical analyses were performed using the Mann Whitney U test for panels **(A,D)**, and the Kruskal Wallis test for panel **(C)**. Post hoc testing was not performed because no significant difference was detected in the Kruskal Wallis test. *p < 0.05, ****p < 0.0001; ns, not significant.

Because HHV-6B SITH-1 expression can be inferred from anti-SITH-1 antibody titers in peripheral blood (Kobayashi et al., 2020), we compared these titers between patients with PASC and normal controls (NCs). A total of 156 individuals with PASC were evaluated alongside 36 NCs with no history of SARS-CoV-2 infection (Table 1). Anti-SITH-1 antibody titers were significantly higher in the PASC group (Figure 1A). Titers were normalized to the median value observed in NCs, which was set to 1. The Receiver operating characteristic (ROC) analysis yielded an AUC of 0.819 (95% CI, 0.759–0.879). Using the threshold of 1.43, sensitivity was 62.8% (95% CI, 55.0%–70.0%) and specificity was 91.7% (95% CI, 78.2%–97.1%).

Receiver operating characteristic (ROC) analysis revealed that, when a threshold of 1.43 was applied, 62.8% of PASC patients tested positive for anti-SITH-1 antibodies, compared to 5.6% of NCs (Figure 1B). To explore the possible impact of different SARS-CoV-2 variants, PASC patients were categorized according to the presumed infecting strain (wild type, α, δ, or ο), based on the period of infection. No significant differences in anti-SITH-1 antibody titers were observed among these groups (Figure 1C).

Furthermore, among the most prevalent symptoms reported after COVID-19, fatigue and depression were significantly more severe in patients who were seropositive for anti-SITH-1 antibodies compared to those who were seronegative (Figure 1D). The severity of symptoms was assessed using the highest numerical rating scale (NRS) score during the observation period.

### 3.2 Mechanistic investigation of HHV-6B reactivation and SITH-1–induced neuropsychiatric symptoms using a mouse model

**FIGURE 2:**
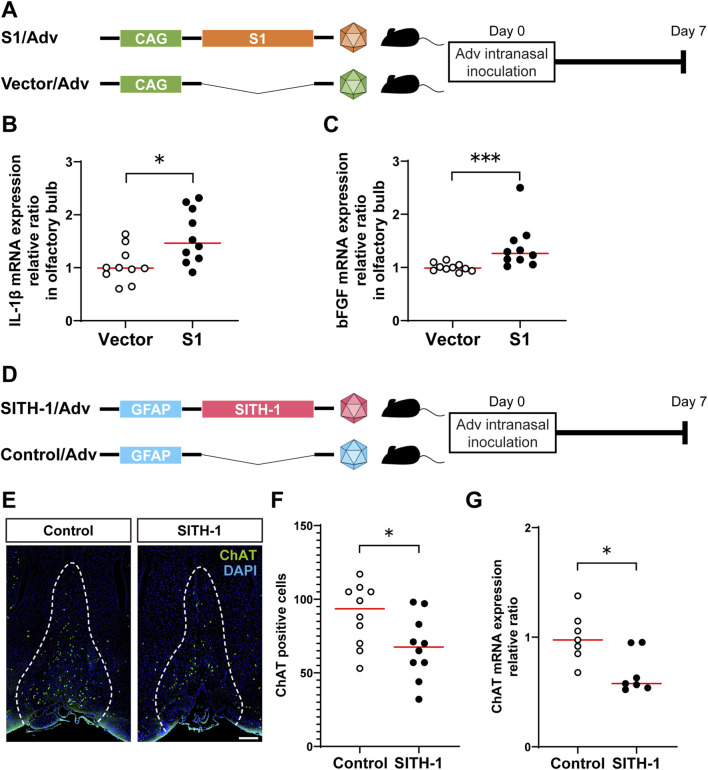
Mechanistic investigation of HHV-6B reactivation and SITH-1 induced cholinergic deficits using mouse models. **(A)** Schematic illustration of the adenoviral vectors S1/Adv and Vector/Adv, and the experimental design. Mice were analyzed 7 days after intranasal inoculation with each adenoviral vector. **(B)** IL-1β mRNA expression in the olfactory bulb of mice inoculated with S1 expressing adenovirus or control vector. Group sizes were as follows: Vector (n = 10), S1 (n = 10). Data represent pooled results from two independent experiments (5 mice per group per experiment). **(C)** bFGF mRNA expression in the olfactory bulb of mice inoculated with S1 expressing adenovirus or control vector. Group sizes were as follows: Vector (n = 10), S1 (n = 10). Data represent pooled results from two independent experiments (5 mice per group per experiment). **(D)** Schematic illustration of the adenoviral vectors SITH-1/Adv and Control/Adv, and the experimental design. Mice were analyzed 7 days after intranasal inoculation with each adenoviral vector. **(E)** Representative immunofluorescence images of choline acetyltransferase (ChAT) in the medial septum (MS) and diagonal band of Broca (DBB) (green, ChAT; blue, DAPI; scale bar, 200 μm). **(F)** Quantification of the number of ChAT positive cells in the MS and DBB in mice transiently expressing SITH-1 compared with control mice. Group sizes were as follows: Control (n = 10), SITH-1 (n = 10). Data represent pooled results from two independent experiments (5 mice per group per experiment). **(G)** ChAT mRNA expression in the whole brain in mice transiently expressing SITH-1 compared with control mice. Group sizes were as follows: Control (n = 7), SITH-1 (n = 7). Data represent pooled results from two independent experiments (3-4 mice per group per experiment). Red horizontal lines indicate median values. Statistical comparisons for panels **(B,C,F,G)** were performed using the Mann-Whitney U test. ∗, p < 0.05; ∗∗∗, p < 0.001.

To investigate the mechanisms underlying HHV-6B reactivation and the exacerbation of neuropsychiatric symptoms observed in PASC patients, we employed a mouse model. SITH-1 is known to be expressed during HHV-6B reactivation in latently infected astrocytes of the olfactory bulb (Kobayashi et al., 2020), and previous studies have identified interleukin-1β (IL-1β) and basic fibroblast growth factor (bFGF) as reactivation-inducing factors in these cells (Tamai et al., 2017). In a mouse model in which the SARS-CoV-2 spike (S1) protein was transiently expressed in the nasal cavity, mimicking viral exposure in human COVID-19 (Figure 2A), we observed upregulated expression of IL-1β (Figure 2B) and bFGF (Figure 2C) in the olfactory bulb. These findings suggest that SARS-CoV-2 S1 protein may contribute to HHV-6B reactivation and subsequent SITH-1 expression in the olfactory bulb of PASC patients.

To further explore the relationship between HHV-6B SITH-1 and PASC-associated neuropsychiatric symptoms, we created a mouse model in which SITH-1 was transiently expressed in olfactory bulb astrocytes via intranasal administration of an adenoviral vector encoding the HHV-6B SITH-1 gene (hereafter referred to as the SITH-1 mouse) (Figure 2D). Consistent with previous reports, these mice exhibited olfactory bulb apoptosis and depression-like behaviors (Kobayashi et al., 2020). Given that nasal expression of the SARS-CoV-2 S1 protein in mice has been shown to reduce acetylcholine production and induce neuropsychiatric abnormalities (Oka et al., 2023), we examined whether SITH-1 expression induces similar cholinergic deficits. Immunohistochemical analysis revealed a marked reduction in choline acetyltransferase (ChAT), the key enzyme for acetylcholine synthesis, in the medial septum (MS) and diagonal band of Broca (DBB) (Figures 2E,F), along with decreased ChAT expression in the whole brain (Figure 2G). These findings suggest that SITH-1 expressed as a consequence of HHV-6B reactivation induced by SARS-CoV-2 infection, similar to the SARS-CoV-2 S1 protein, reduces acetylcholine production and thereby contributes to the development of neuropsychiatric symptoms.

### 3.3 Donepezil ameliorates neuropsychiatric symptoms in the SITH-1 mouse model

**FIGURE 3:**
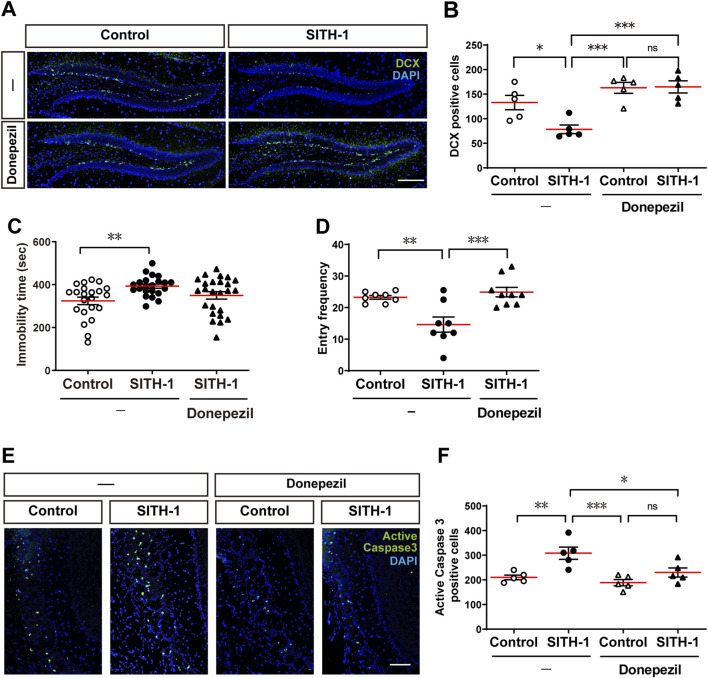
Donepezil ameliorates neuropsychiatric phenotypes in the SITH-1 mouse model. **(A)** Representative immunofluorescence images of doublecortin (DCX)-positive cells in the hippocampus in mice treated with Control/Adv or SITH-1/Adv, with or without donepezil (green, DCX; blue, DAPI; scale bars, 200 μm). **(B)** Quantification of DCX-positive cells in the hippocampus. n = 5 mice per group. **(C)** Immobility time in the tail suspension test. Group sizes were as follows: Control + no drug (n = 22), SITH-1 + no drug (n = 22), SITH-1 + Donepezil (n = 25). Data represent pooled results from five independent experiments (3-5 mice per group per experiment). **(D)** Number of arm entries in the Y-maze test. Group sizes were as follows: Control + no drug (n = 8), SITH-1 + no drug (n = 8), SITH-1 + Donepezil (n = 9). Data represent pooled results from two independent experiments (3-5 mice per group per experiment). **(E)** Representative immunofluorescence images of active caspase-3-positive cells in the olfactory bulb in mice treated with Control/Adv or SITH-1/Adv, with or without donepezil (green, active caspase-3; blue, DAPI; scale bars, 100 μm). **(F)** Quantification of active caspase-3-positive cells in the olfactory bulb. n = 5 mice per group. Values are presented as mean ± SEM. One-way ANOVA followed by Fisher’s LSD *post hoc* test. *, p < 0.05; **, p < 0.01; ***, p < 0.001; ns, not significant.

The efficacy of the acetylcholinesterase inhibitor donepezil in ameliorating neuropsychiatric symptoms induced by SITH-1 was evaluated using the SITH-1 mouse model. In the SITH-1 mouse model, reduced hippocampal neurogenesis was observed. This reduction was reversed following donepezil administration (Figures 3A,B), suggesting its potential to mitigate SITH-1–induced impairments in memory and depressive behavior.

Behavioral testing revealed that SITH-1 mice exhibited prolonged immobility time in the tail suspension test, indicative of depression-like behavior, and a reduced frequency of entries in the Y-maze, reflecting decreased spontaneous activity. Both phenotypes were significantly improved by donepezil treatment (Figures 3C,D). In addition, olfactory bulb apoptosis, which likely contributes to decreased central acetylcholine production in this model, was also ameliorated by donepezil administration (Figure 3E). These findings collectively support the therapeutic potential of donepezil in alleviating SITH-1–induced neuropsychiatric symptoms.

### 3.4 Donepezil reduces fatigue and depressive symptoms in PASC patients with anti-SITH-1 antibody positivity

Based on the preclinical findings, we hypothesized that donepezil may exert therapeutic effects in PASC patients with elevated anti-SITH-1 antibody titers. To test this, we conducted a subgroup analysis within a previously reported randomized, double-blind clinical trial of donepezil in post-COVID-19 patients (Kawabata et al., 2024; Nakamura et al., 2025), stratifying participants based on their anti-SITH-1 antibody status. The threshold for antibody positivity was defined according to the ROC analysis shown in Figures 1A,B.

**FIGURE 4:**
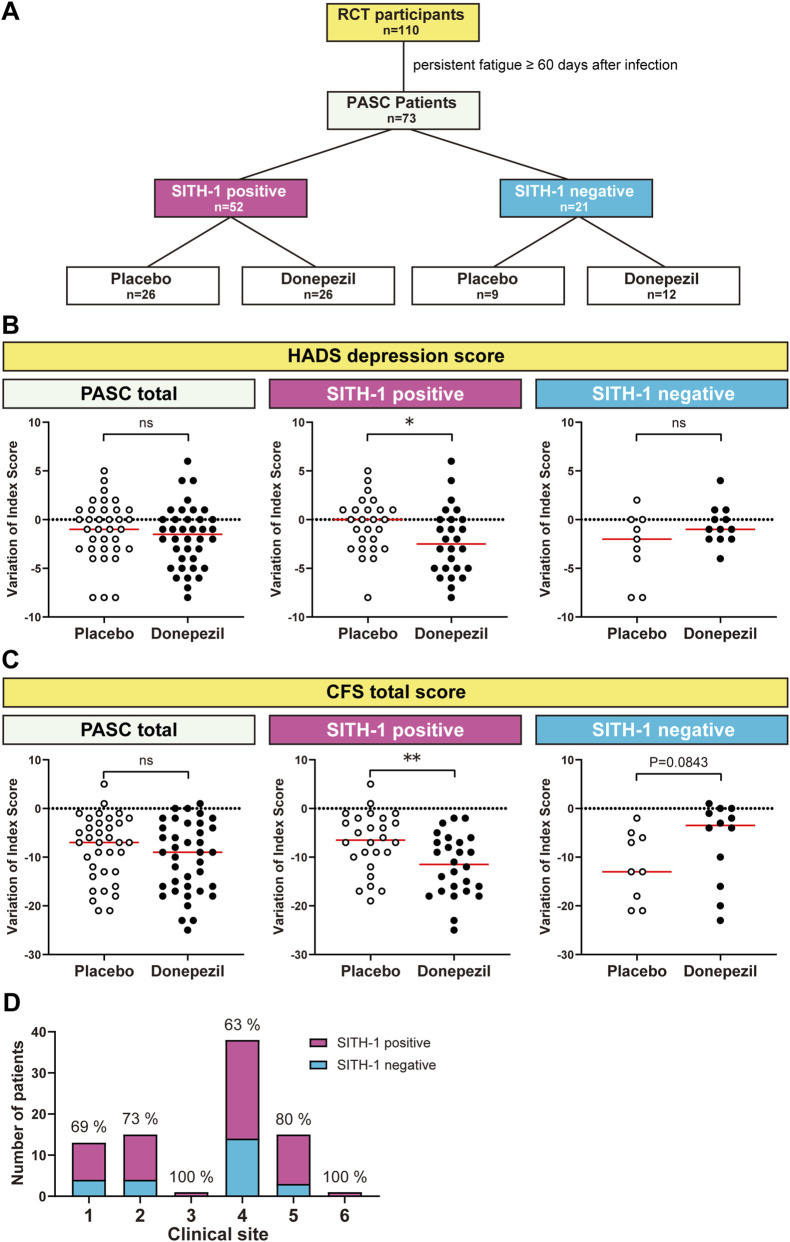
Subgroup analysis of a donepezil randomized clinical trial using anti-SITH-1 antibody titers as a biomarker. **(A)** Flow diagram of participants included in the retrospective subgroup analysis. Among 110 RCT participants, 73 were included as PASC patients based on the operational definition used in this study, defined as persistent fatigue lasting at least 60 days after SARS CoV 2 infection. These 73 patients were stratified by anti-SITH-1 antibody status (positive n = 52, negative n = 21). Within each stratum, participants were analyzed according to their original randomized treatment assignment to placebo or donepezil (anti SITH-1 antibody positive, placebo n = 26, donepezil n = 26; anti SITH-1 antibody negative, placebo n = 9, donepezil n = 12). **(B)** Change from baseline in HADS depression total score at week 3. Three comparisons are shown: all PASC patients included in the subgroup analysis (n = 73, left), anti-SITH-1 antibody positive patients (n = 52, middle), and anti-SITH-1 antibody negative patients (n = 21, right). **(C)** Change from baseline in Chalder Fatigue Scale (CFS) total score at week 8. Three comparisons are shown: all PASC patients included in the subgroup analysis (n = 73, left), anti-SITH-1 antibody positive patients (n = 52, middle), and anti-SITH-1 antibody negative patients (n = 21, right). The p value for the comparison in anti-SITH-1 antibody negative patients is shown in the figure. **(D)** Numbers and percentages of anti-SITH-1 antibody positive participants by clinical site. For **(B)** and **(C)**, change from baseline was calculated as the total score at week 3 or week 8 minus the baseline total score, with more negative values indicating greater improvement. Red horizontal lines indicate median values. Statistical comparisons between placebo and donepezil groups were performed using the Mann Whitney U test. *, p < 0.05; **, p < 0.01; ns, not significant.

From among the 110 participants enrolled in the original clinical trial (Nakamura et al., 2025), we identified a subgroup of 73 patients who met the WHO criteria for PASC, defined as persistent fatigue lasting at least 2 months following SARS-CoV-2 infection (Figure 4A). Baseline characteristics, including age, sex, and post-onset period, did not differ significantly between the donepezil and placebo groups in the overall cohort, nor when stratified by anti-SITH-1 antibody status (Table 2). Fatigue was assessed using the 11-item Chalder Fatigue Scale (CFS) (Morriss et al., 1998; Jing et al., 2016), while depressive symptoms were evaluated using the Patient Health Questionnaire-9 (PHQ-9) (Kroenke et al., 2010; Costantini et al., 2021) and the Hospital Anxiety and Depression Scale (HADS) (Fernández-de-Las-Peñas et al., 2022). Each scale consists of multiple items scored from 0 to 3, with total scores used for analysis. Treatment efficacy was quantified by calculating the change in total scores before and after the 3-week treatment period, with more negative values indicating greater improvement. Clinical assessments were conducted at 3 and 8 weeks after treatment initiation.

In anti-SITH-1 antibody positive PASC patients, donepezil significantly reduced HADS depression scores at week 3 compared to placebo (Figure 4B) and CFS total scores at week 8 (Figure 4C). Item-level changes in the HADS depression subscale at week 3 and the CFS at week 8 are shown in Supplementary Figures S1, S2, respectively. No significant improvement was observed in PHQ-9 scores. In addition to the outcomes shown in Figure 4, results for the remaining questionnaire outcomes and time points are presented in Supplementary Figure S3. Nominal p-values and BH-FDR-adjusted q-values for all placebo versus donepezil comparisons across questionnaires, strata, and time points are summarized in Supplementary Table S1. Across clinical sites, the proportion of patients positive for anti-SITH-1 antibodies ranged from 63.2% to 100%, with an overall positivity rate of 71.7% (Figure 4D). Together, these findings suggest that donepezil is effective in alleviating neuropsychiatric symptoms in PASC patients who are positive for anti-SITH-1 antibodies.

## 4 Discussion

To identify a biomarker for subgroup analysis in our randomized clinical trial (RCT) of donepezil, we measured anti-SITH-1 antibody titers in 156 patients with PASC and in healthy individuals with no history of SARS-CoV-2 infection. Anti-SITH-1 antibody positivity was observed in 62.8% of PASC patients (Figures 1A,B). Given that the positivity rate in the general population is typically around 25% (Kobayashi et al., 2020), these findings suggest that the elevated antibody levels in PASC likely result from HHV-6B reactivation triggered by SARS-CoV-2 infection. Reactivation of latent HHV-6B in olfactory bulb astrocytes has been reported to be induced by interleukin-1β (IL-1β) and basic fibroblast growth factor (bFGF). Consistent with these findings, our current study demonstrated increased expression of IL-1β and bFGF in the olfactory bulb of mice in which the SARS-CoV-2 spike (S1) protein was expressed in the nasal cavity (Figures 2A–C). Since S1 protein expression in the nasal cavity has been frequently observed in COVID-19 patients (Bilinska et al., 2020; Brann et al., 2020; Hoffmann et al., 2020; Shang et al., 2020), these results suggest that SITH-1 expression in the olfactory bulb of PASC patients may arise from HHV-6B reactivation induced by SARS-CoV-2 infection.

The SARS-CoV-2 S1 protein has been implicated in the neuropsychiatric complications associated with COVID-19 and PASC (Olajide et al., 2022; Oka et al., 2023; Chang et al., 2024). In previous work, a mouse model with nasal S1 protein expression (S1 mouse) was shown to exhibit olfactory bulb damage, which led to reduced central acetylcholine production. This, in turn, disrupted the cholinergic anti-inflammatory pathway, resulting in heightened brain inflammation and ultimately leading to neuropsychiatric symptoms such as depression (Oka et al., 2023). In the current study, we investigated whether HHV-6B SITH-1 expression in the olfactory bulb similarly affects acetylcholine production. In SITH-1 mice, we observed a decrease in brain acetylcholine levels (Figures 2E–G), suggesting that SITH-1 contributes to cholinergic dysfunction. Since olfactory S1 expression has been widely documented in COVID-19 patients (Bilinska et al., 2020; Brann et al., 2020; Hoffmann et al., 2020; Shang et al., 2020), and anti-SITH-1 antibody positivity indicates SITH-1 expression in the olfactory bulb, it is plausible that HHV-6B reactivation initiated by S1 may also sustain acetylcholine suppression via SITH-1, prolonging neuropsychiatric symptoms in PASC. This mechanism may partially explain why PASC patients with anti-SITH-1 antibody positivity exhibit more severe fatigue and depressive symptoms, as observed in our initial clinical cohort (Figure 2).

We further assessed the therapeutic potential of the acetylcholinesterase inhibitor donepezil in SITH-1 mice. These mice exhibited hippocampal neurogenesis deficits and depression-like behaviors, both of which were ameliorated by donepezil treatment (Figures 3A–D). Additionally, olfactory bulb apoptosis, which likely underlies reduced acetylcholine production, was also improved by donepezil (Figures 3E,F). These findings support the notion that donepezil may effectively target SITH-1 induced neuropsychiatric dysfunction. On the other hand, the SITH-1 mouse model relies on transient adenoviral expression in the olfactory bulb and does not fully recapitulate the complexity of HHV-6B latency and reactivation in humans. Mouse behavioral readouts should be interpreted as domain-specific proxies, and prospective validation in human cohorts and additional models is warranted.

In our subgroup analysis of the clinical trial, donepezil significantly improved fatigue and depressive symptoms in patients with PASC who were seropositive for anti-SITH-1 antibodies, who accounted for 71.7% of the PASC cohort. In contrast, in seronegative individuals, donepezil tended to worsen symptoms. This may be due to its well-known gastrointestinal side effects (Bryson and Benfield, 1997; Takeda et al., 2006). Patients in the seronegative group who did not respond to donepezil may have perceived greater overall symptom burden due to these side effects. These results suggest that combining anti-SITH-1 antibody testing with donepezil administration may enable a companion diagnostic strategy for PASC-related fatigue. Because this subgroup analysis was conducted *post hoc*, the results should be interpreted as exploratory and hypothesis-generating. Nevertheless, the treatment signal was observed mainly in anti-SITH-1-positive patients and was directionally consistent across key outcomes, supporting the hypothesis that donepezil may be beneficial in a biomarker-defined subset of PASC patients. These findings warrant prospective validation in a biomarker-stratified trial.

The lack of a significant improvement in PHQ-9 despite improvements in HADS depression may reflect differences in the symptom domains emphasized by these instruments. HADS depression places greater weight on reduced interest and anhedonia-related features, whereas PHQ-9 captures a broader range of DSM-based depressive symptoms, including sleep and appetite disturbance, psychomotor changes, and suicidal ideation. Because cholinergic signaling has been implicated in reward-related and motivational processes (Wang et al., 2021), cholinergic augmentation with donepezil could preferentially affect symptom domains more strongly reflected by HADS depression. In addition, donepezil-associated adverse effects, particularly gastrointestinal symptoms, may have attenuated improvements on broader self-reported measures such as PHQ-9.

Considering the impact of fatigue and depression on QOL and work productivity in PASC patients, and the fact that approximately two-thirds are seropositive for anti-SITH-1 antibodies, donepezil may offer clinically meaningful benefits to a substantial portion of this population.

Recent studies have highlighted the association between fatigue, depression, and brain inflammation (Maes et al., 2012; Nakatomi et al., 2014; Morris et al., 2016), and brain inflammation has also been reported in patients with PASC (Peluso et al., 2022; VanElzakker et al., 2024). In our previous animal study, we demonstrated that donepezil alleviated fatigue- and depression-like symptoms by suppressing brain inflammation through restoration of the cholinergic anti-inflammatory pathway (Oka et al., 2023). In the present study, our findings suggest that the expression of SITH-1 in the brain may contribute to the development of brain inflammation by reducing acetylcholine production. Donepezil may help alleviate fatigue and depressive symptoms in humans by modulating brain inflammation through restoration of the cholinergic anti-inflammatory pathway.

Overall, our findings suggest that SITH-1 expression resulting from HHV-6B reactivation contributes to neuropsychiatric complications, such as fatigue and depressive symptoms, in approximately two-thirds of PASC patients. Targeting cholinergic dysfunction with agents such as donepezil may therefore offer an effective treatment strategy for SITH-1–associated PASC (Figure 4). Measuring anti-SITH-1 antibody titers may serve as a valuable companion diagnostic to guide donepezil therapy in clinical practice.

### 4.1 Limitations of the study

Anti-SITH-1 antibodies were not measured prior to developing COVID-19 and associations with HHV-6B reactivation and SITH-1 expression in patients developing PASC were not tracked over time. Also, since almost all adults are latently infected with HHV-6B, we could not perform a study on a population not infected by HHV-6B. Because the subgroup analysis was exploratory and not pre-specified, the findings require confirmation in a prospective biomarker-stratified trial. Accordingly, the subgroup findings should be interpreted as hypothesis-generating, with full outcome results and BH-FDR-adjusted q-values provided in Supplementary Figure S3; Supplementary Table S1. The ROC-derived threshold was developed in the present cohort and has not been externally validated; therefore, its generalizability requires confirmation. Vaccination status was not systematically available for the RCT cohort. In the observational cohort, data on acute COVID-19 severity, vaccination status, comorbid psychiatric conditions, and concomitant medications were not uniformly collected, which may allow residual confounding.

## Data Availability

The original contributions presented in the study are included in the article/Supplementary Material, further inquiries can be directed to the corresponding author.
