# Supplementary material for: Donepezil ameliorates fatigue and depression in PASC patients with HHV-6B SITH-1-induced acetylcholine deficiency
Source: Front Pharmacol. 2026 Jun 4;17:1807203. doi: 10.3389/fphar.2026.1807203 (PMC13275412; doi:10.3389/fphar.2026.1807203)
Supplement: Supplementary file 1 [file Supplementaryfile1.docx]

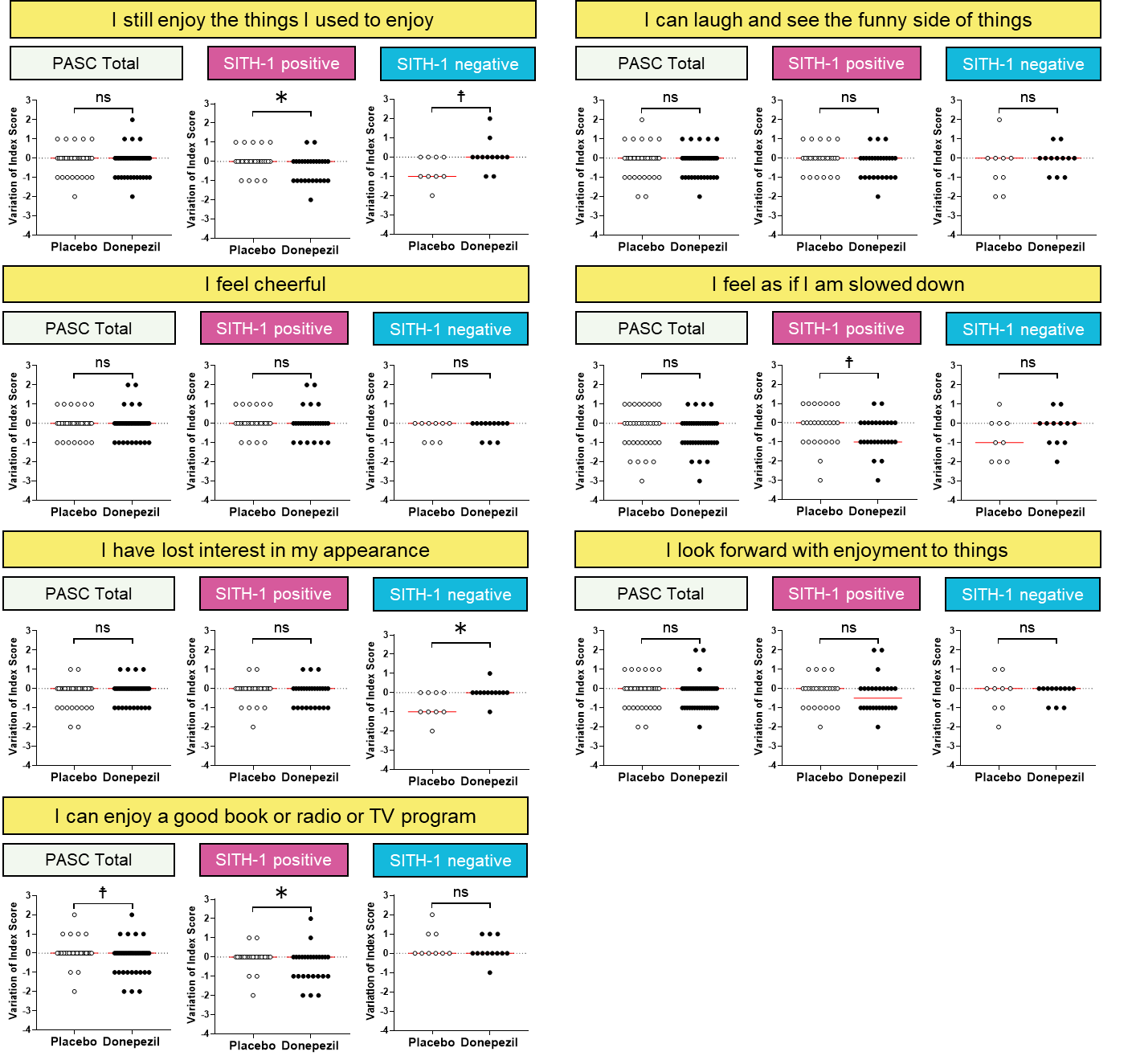


**Supplementary Figure S1. Changes in individual items of the HADS depression subscale at week 3.**

Change from baseline was calculated as the item score at week 3 minus the baseline item score, with more negative values indicating greater improvement. Three comparisons are shown for each item: all PASC patients included in the subgroup analysis (n = 73, left), anti-SITH-1 antibody-positive patients (n = 52, middle), and anti-SITH-1 antibody-negative patients (n = 21, right). Red horizontal lines indicate median values. Statistical comparisons between placebo and donepezil groups were performed using the Mann–Whitney U test. ☨p < 0.1; *p < 0.05; ns, not significant.


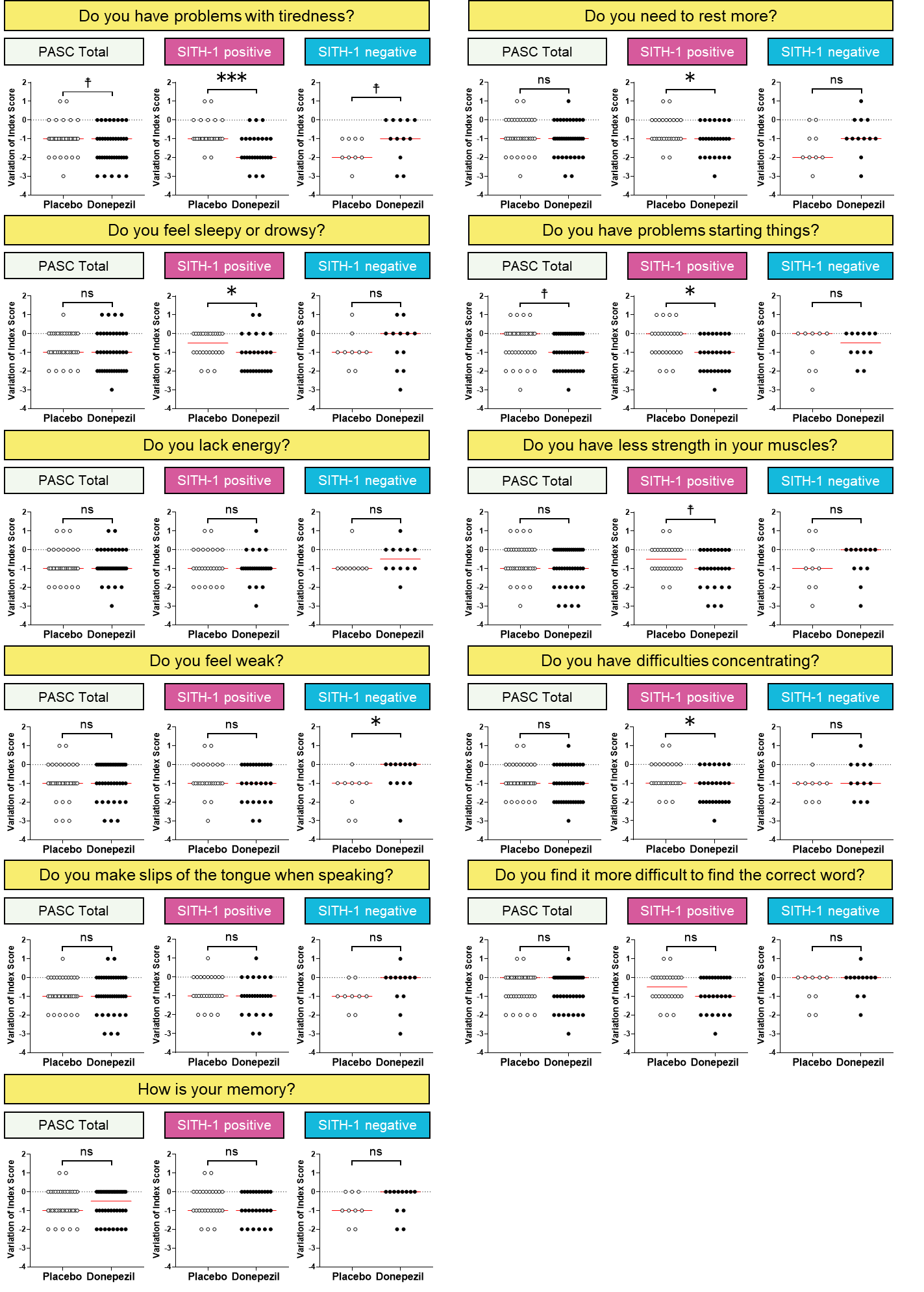


**Supplementary Figure S2. Changes in individual items of the Chalder Fatigue Scale at week 8.**

Change from baseline was calculated as the item score at week 8 minus the baseline item score, with more negative values indicating greater improvement. Three comparisons are shown for each item: all PASC patients included in the subgroup analysis (n = 73, left), anti-SITH-1 antibody-positive patients (n = 52, middle), and anti-SITH-1 antibody-negative patients (n = 21, right). Red horizontal lines indicate median values. Statistical comparisons between placebo and donepezil groups were performed using the Mann–Whitney U test. ☨p < 0.1; *p < 0.05; ***p < 0.001; ns, not significant.


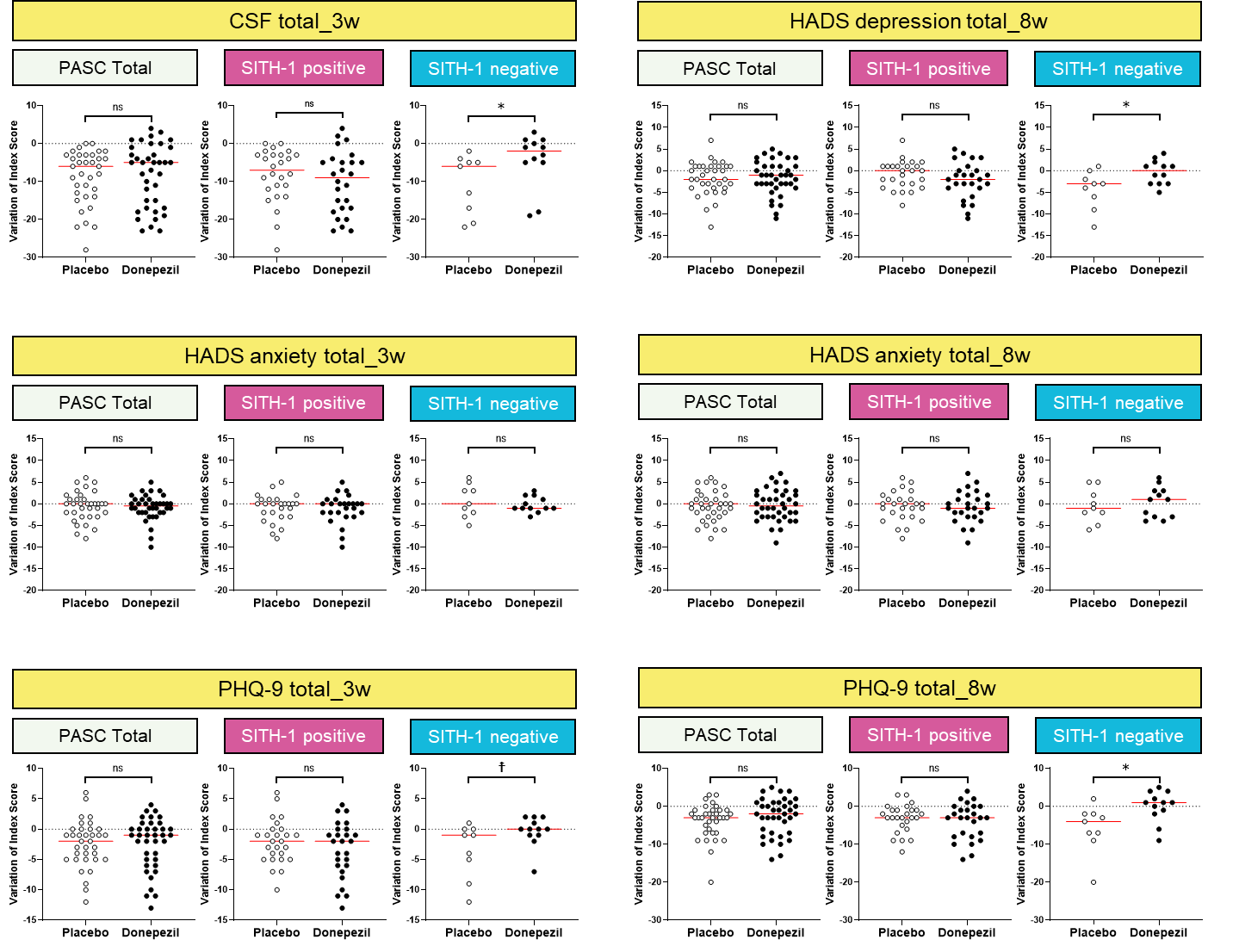


**Supplementary Figure S3. Exploratory subgroup comparisons for questionnaire outcomes not shown in Figure 4.**

Change from baseline was calculated as the total score at week 3 or week 8 minus the baseline total score, with more negative values indicating greater improvement. Three comparisons are shown for each panel: all PASC patients included in the subgroup analysis (n = 73, left), anti-SITH-1 antibody-positive patients (n = 52, middle), and anti-SITH-1 antibody-negative patients (n = 21, right). Red horizontal lines indicate median values. Statistical comparisons between placebo and donepezil groups were performed using the Mann–Whitney U test. ☨p < 0.1; *p < 0.05; ns, not significant.

| Outcome | Time point | Stratum | Placebo  (n) | Donepezil  (n) | p value | q value |
| --- | --- | --- | --- | --- | --- | --- |
| CFS total | Week 3 | PASC Total | 35 | 38 | 0.698 | 0.698 |
|  |  | SITH-1 positive | 26 | 26 | 0.288 | 0.346 |
|  |  | SITH-1 negative | 9 | 12 | 0.023* | 0.069^☨^ |
|  | Week 8 | PASC Total | 35 | 38 | 0.279 | 0.346 |
|  |  | SITH-1 positive | 26 | 26 | 0.009** | 0.054^☨^ |
|  |  | SITH-1 negative | 9 | 12 | 0.084^☨^ | 0.169 |
| HADS depression total | Week 3 | PASC Total | 35 | 38 | 0.256 | 0.307 |
|  |  | SITH-1 positive | 26 | 26 | 0.037* | 0.110 |
|  |  | SITH-1 negative | 9 | 12 | 0.220 | 0.307 |
|  | Week 8 | PASC Total | 35 | 38 | 0.884 | 0.884 |
|  |  | SITH-1 positive | 26 | 26 | 0.248 | 0.307 |
|  |  | SITH-1 negative | 9 | 12 | 0.028* | 0.110 |
| HADS anxiety total | Week 3 | PASC Total | 35 | 38 | 0.758 | 0.872 |
|  |  | SITH-1 positive | 26 | 26 | 0.872 | 0.872 |
|  |  | SITH-1 negative | 9 | 12 | 0.632 | 0.872 |
|  | Week 8 | PASC Total | 35 | 38 | 0.862 | 0.872 |
|  |  | SITH-1 positive | 26 | 26 | 0.830 | 0.872 |
|  |  | SITH-1 negative | 9 | 12 | 0.659 | 0.872 |
| PHQ-9 total | Week 3 | PASC Total | 35 | 38 | 0.649 | 0.698 |
|  |  | SITH-1 positive | 26 | 26 | 0.441 | 0.661 |
|  |  | SITH-1 negative | 9 | 12 | 0.067^☨^ | 0.201 |
|  | Week 8 | PASC Total | 35 | 38 | 0.217 | 0.433 |
|  |  | SITH-1 positive | 26 | 26 | 0.698 | 0.698 |
|  |  | SITH-1 negative | 9 | 12 | 0.014* | 0.081^☨^ |

**Supplementary Table S1. Exploratory subgroup comparisons across questionnaire outcomes with BH-FDR adjustment**

Nominal p values for placebo versus donepezil comparisons in the exploratory post hoc subgroup analyses are shown, together with Benjamini–Hochberg false discovery rate (BH-FDR) adjusted q values. Comparisons were performed within each stratum (overall PASC, anti-SITH-1-positive PASC, anti-SITH-1-negative PASC) at week 3 and week 8. For each questionnaire, BH-FDR adjustment was applied within that questionnaire family across six comparisons (three strata by two time points). P values were obtained using the Mann–Whitney U test. ☨p < 0.1; *p < 0.05; **p < 0.01. P values are nominal; q values are BH-FDR adjusted within each questionnaire family. ☨q < 0.1.
